# The Potential Roles of IL-1β, IL-6, and RIPK3 in the Pathogenesis of Stevens–Johnson Syndrome/Toxic Epidermal Necrolysis

**DOI:** 10.3390/diagnostics15030290

**Published:** 2025-01-26

**Authors:** Chandana Sooranahalli, Vidhya R. Rao, Brandon Zelman, Mallika Shekhar, Sevnur Komurlu Keceli, Charles Bouchard, Omer Iqbal

**Affiliations:** 1 Department of Ophthalmology, Stritch School of Medicine, Loyola University Chicago, Maywood, IL 60153, USA; csooranahalli@luc.edu (C.S.); mshekhar@luc.edu (M.S.); 2 Department of Ophthalmology, Loyola University Medical Center, Maywood, IL 60153, USA; vrao2@luc.edu (V.R.R.); cboucha@lumc.edu (C.B.); 3 Department of Pathology, Loyola University Medical Center, Maywood, IL 60153, USA; brandon.zelman@luhs.org; 4 Department of Microbiology & Immunology, Stritch School of Medicine, Loyola University Chicago, Maywood, IL 60153, USA; skomurlu@luc.edu; 5 Departments of Ophthalmology & Pathology, Stritch School of Medicine, Loyola University Chicago, Maywood, IL 60153, USA

**Keywords:** Stevens–Johnson syndrome (SJS), (TEN), SJS/TEN pathogenesis, severe cutaneous adverse reactions (SCARs), IL-1β, IL-6, RIPK3, cytokines in SJS/TEN, immune response in skin disorders, inflammatory pathways in SJS/TEN

## Abstract

**Background/Objectives:** Stevens–Johnson Syndrome and Toxic Epidermal Necrolysis (SJS/TEN) are rare but severe skin conditions, often triggered by medications, that can be life-threatening. These conditions frequently affect the eyes, causing ocular surface disease, which can result in visual impairment or blindness. Although the exact mechanisms behind SJS/TEN remain unclear, key inflammatory mediators such as IL-1β, IL-6, and RIPK3 are believed to play critical roles in inflammation, necroptosis, and regulatory processes. Investigating these factors offers new insights into the disease’s underlying mechanisms and potential targets for treatment. This study aims to determine the roles of IL-1β, IL-6, and RIPK3 in the pathogenesis of SJS/TEN. **Methods:** The study examined the expression levels of IL-1β, IL-6, and RIPK3 in skin biopsies from patients with biopsy-confirmed SJS/TEN, using lichen planus as a positive control and normal skin as a baseline control. Immunohistochemistry was employed for this analysis. Additionally, the impact of SJS/TEN patient plasma on mitochondrial function was assessed in platelets and human corneal epithelial (H-CET) cells. Using a fluorescent plate reader, mitochondrial activity and superoxide ion levels were measured, comparing plasma from SJS/TEN patients to normal human plasma. **Results:** Skin biopsies from SJS/TEN patients showed a significantly higher expression of IL-1β, IL-6, and RIPK3 compared to both lichen planus and normal controls. Furthermore, plasma from SJS/TEN patients significantly reduced platelet viability and increased mitochondrial and total cellular superoxide ions, as demonstrated by elevated levels of MitoSOX Red and CellROX Red. **Conclusions:** These findings suggest that IL-1β, IL-6, and RIPK3 may contribute to the pathogenesis of SJS/TEN and highlight their potential as targets for therapeutic intervention.

## 1. Introduction

Stevens–Johnson Syndrome (SJS) and Toxic Epidermal Necrolysis (TEN) are generally classified as Severe Cutaneous Adverse Reactions (SCARs), characterized by extensive necrosis and detachment of the epidermis. These rare and life-threatening conditions affect mucocutaneous membranes in over 90% of cases, often leading to complications across multiple organ systems, including hematological, ophthalmological, and genitourinary systems [1]. Patients with SJS/TEN typically present with widespread blistering rashes, high fever, and mucous membrane damage. SJS and TEN exist on a continuum and are classified based on the percentage of body surface area (BSA) involved. The term “epidermal necrolysis” is often used to collectively refer to SJS, SJS/TEN overlap, and TEN.

Both conditions are exceptionally rare, with an incidence rate of approximately five to six cases per million individuals annually, and many patients require hospitalization. The mortality rate for SJS ranges from 1% to 5%, while TEN has a significantly higher mortality rate of 20% to 30%. Survivors frequently face long-term complications, such as vision loss due to pseudo-membrane formation [2].

Examinations of skin sections affected by these conditions show orthokeratosis overlying the remainder of the epidermis with full-thickness necrosis of the keratinocytes. The underlying dermis consists of mild superficial perivascular and interstitial inflammation consisting of lymphocytes and eosinophils, consistent with the clinical diagnosis of SJS.

The pathogenesis of epidermal necrolysis is not fully understood. Case studies have strongly linked SJS/TEN to specific medications, with symptoms typically appearing 1–4 weeks after starting a new drug. Over 200 medications have been implicated, including anti-gout agents, antibiotics, antipsychotics, antiepileptics, and nonsteroidal anti-inflammatory drugs (NSAIDs) [2]. Although the overall risk is low, genetic predispositions significantly increase susceptibility. Strong associations have been documented between specific HLA genotypes and SJS/TEN in certain populations, such as HLA-B1502 with carbamazepine-induced SJS/TEN in Han Chinese, Thai, and Malay populations, and HLA-B5801 with allopurinol-induced SJS/TEN in Han Chinese, Malay, Thai, European, and Korean populations [3,4,5,6]. In Caucasians, HLA-A31:01 and HLA-B11:01 have been linked to carbamazepine-induced SJS/TEN [7].

SJS/TEN are believed to be immune-mediated hypersensitivity reactions triggered by cytotoxic CD8+ T lymphocytes. The activation of these cells and the subsequent release of cytotoxic proteins lead to widespread epidermal necrolysis. Additional studies have shown infiltration of natural killer (NK) cells and T-helper type 17 (Th17) cells in skin lesions. In TEN, T lymphocytes at lesion sites exhibit drug-specific cytotoxicity against allogeneic cells sharing the same HLA as autologous cells [8].

The secondary activation of cytokine cascades may contribute to clinical variability. Inflammatory cytokines such as TNF-α and its receptor TNF receptor 1 are reported in cases of SJS/TEN [1,9,10]. Granulysin, a cytolytic protein produced by cytotoxic T cells, NK/T cells, and NK cells, is now recognized as a critical mediator of keratinocyte death in SJS/TEN [8]. Recent studies from our laboratories have explored the roles of IL-13, IL-15, IL-33, TGF-β, NKG2C, and NLRP3 in SJS/TEN pathogenesis, providing new insights into the complex immune mechanisms underlying these conditions [7,8,11,12,13,14,15].

Identifying unique biomarkers is crucial for understanding the mechanisms of action of SJS/TEN. Interleukin-1β (IL-1β) is a central regulator of inflammation, promoting cytokine cascades, immune cell activation, and apoptosis. Elevated IL-1β has been linked to inflammatory diseases, autoimmune disorders, and certain cancers. It is produced by monocytes, macrophages, Langerhans cells, dendritic cells, and keratinocytes. Blocking IL-1β activity is emerging as a therapeutic strategy [16].

Interleukin-6 (IL-6) is a multifunctional cytokine with both pro- and anti-inflammatory properties. It is upregulated in SJS/TEN and correlates with disease severity and cutaneous involvement [17,18,19]. Higher plasma levels of IL-6 have been observed in TEN patients compared to controls, implicating its role in systemic inflammation and tissue damage [20].

The predominant mechanism of cell death in Toxic Epidermal Necrolysis (TEN) is believed to be apoptosis [21]. However, emerging studies suggest that necroptosis—a form of programmed cell death characterized by necrosis and inflammation—may also play a significant role. Receptor-interacting protein kinase 3 (RIPK3), encoded by the RIPK3 gene in humans, is a key enzyme involved in necroptosis, pro-inflammatory gene expression, and sustained translation [22,23,24]. The Ripoptosome complex, which includes RIPK3 and RIPK1, plays a critical role in keratinocyte cell death pathways. The sensitization of keratinocytes to RIPK3-mediated necrosis may exacerbate skin inflammation, contributing to inflammatory skin diseases and chronic inflammation. Notably, RIPK3 expression is highly upregulated in skin biopsies from TEN patients, suggesting that it may drive pathological damage in TEN through the activation of programmed necrotic cell death [25].

Mitochondrial damage also appears to be a crucial factor in the pathogenesis of drug-induced toxicity and TEN [26]. Studies have identified a strong association between mitochondrial dysfunction and inflammasome activation. The generation of mitochondrial reactive oxygen species (ROS) is thought to activate the NLRP3 inflammasome, leading to the production of interleukin-1β (IL-1β). However, autophagic clearance of damaged mitochondria serves to limit this process [27]. Furthermore, mitochondrial ROS may amplify inflammation by inducing the production of interleukin-6 (IL-6) and tumor necrosis factor (TNF) through positive feedback from IL-1β [28]. Additionally, mitochondrial ROS may promote RIPK3 recruitment into the necrosome via RIPK1 autophosphorylation, further contributing to necroptosis and inflammation [29].

Understanding the roles of IL-1β, IL-6, and RIPK3 in SJS/TEN pathogenesis is critical for uncovering novel therapeutic targets. It is hypothesized that these biomarkers, along with mitochondrial dysfunction, contribute to the pathogenesis of SJS/TEN by impairing platelet and human corneal epithelial cell function.

## 2. Methods

### 2.1. Experimental Design for Skin Biopsy Analysis

**Figure 1:**
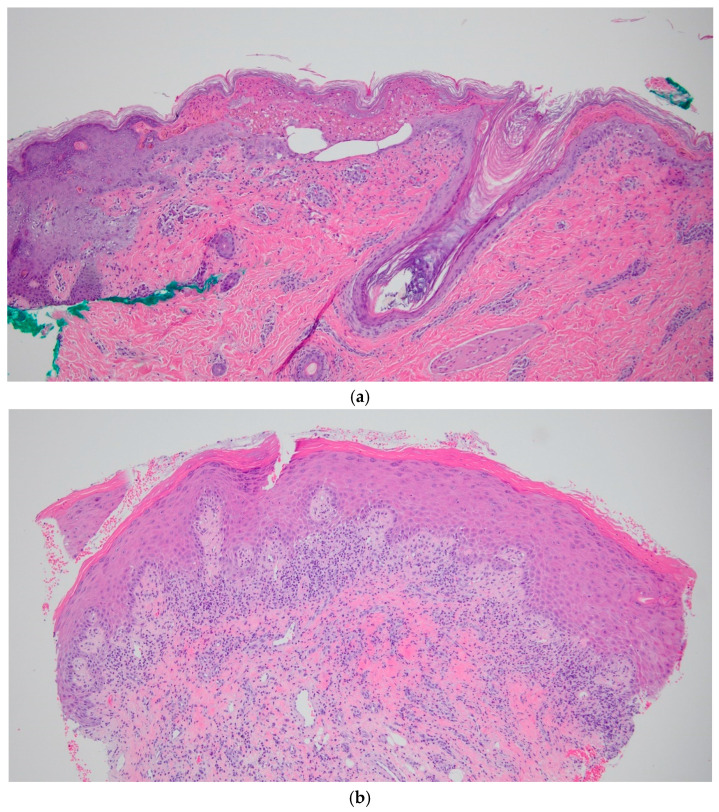
(**a**): Histology sections of SJS patient punch biopsy, stained with H&E at, 10× magnification. (**b**) Histology sections of LP patient punch biopsy, stained with H&E, at 10× magnification.

This study utilized nine archived, unstained slides from biopsy-confirmed SJS/TEN patients (Figure 1a) and nine slides from lichen planus (LP) patients, which served as positive controls (Figure 1b), under a current IRB-approved protocol at Loyola University. The paraffin-embedded skin samples were sourced from the archives of the Loyola University Medical Center core pathology lab. Each sample was sectioned to a thickness of four microns using an American Optical Model 820 microtome and mounted onto positively charged glass slides.

The skin biopsy sections were deparaffinized by washing them three times with xylene (5 min each), followed by sequential washes in 100% ethanol (2 min twice), 95% ethanol (5 min), and 70% ethanol (5 min). After a 1 min rinse with distilled water, the slides were washed in phosphate-buffered saline (PBS) for 5 min. To block nonspecific binding, all slides were treated with normal goat plasma for 1 h. They were then incubated overnight at 4 °C in a humidified dark box with primary antibodies against IL-1β, IL-6, or RIPK3. Following incubation, the slides were washed three times with PBS and treated with secondary anti-rabbit IgG antibodies and diamino-2 phenylindole (DAPI) for nuclear staining for 30 min. After a final PBS wash, the slides were mounted on fluorogel and imaged using a DeltaVision microscope (manufactured by Zeiss Lattice SIM 5 Super Resolution Microscope, Jena, Germany) equipped with a digital camera, maintaining consistent exposure times across all samples.

In addition to the SJS/TEN and LP samples, ten skin biopsy slides from patients without SJS/TEN or any known dermatological conditions served as the normal control group (NC). All slides underwent identical preparation and analysis.

Deconvolution immunofluorescence (IF) was performed on all samples using the DeltaVision microscope. The fluorescent intensity sum per punctum was quantified using Imaris^®^ software (https://imaris.oxinst.com/products/imaris-for-cell-biologists, (accessed on 18 June 2024–Oxford Instruments, Abingdon, UK), after subtracting background autofluorescence. The number of puncta and the intensity of IF above baseline were measured in the 488 nm channel (FITC-stained IL-1β, IL-6, and RIPK3) using the surface function. Statistical analyses were conducted using GraphPad Prism software v10.

### 2.2. Experimental Design for Mitochondrial Functioning Assays

Platelets:

From a normal human control, approximately 100,000 platelets were suspended in 0.1 mL of RPMI media, were plated in 96 well plates (clear bottom, NUNC) previously coated with Poly-D-Lysine (50 µg/mL) and allowed to rest for 30 min. The platelets were then incubated with MitoSox Red (manufactured by ThermoFischer Scientific, Waltham, MA, USA) (1 µM) for 30 min and treated with either normal plasma, SJS plasma, or Innovin (manufactured by Siemens Healthineers Dade, Newark, DE, USA). Innovin (a thromboplastin reagent) and rhTF are commonly used to trigger the extrinsic pathway of coagulation. This treatment was used to assess platelet activation in response to tissue factors and served as an additional negative control to ensure the specificity of the observed response. The changes in MitoSOX Red fluorescence were measured kinetically with a fluorescence plate reader (Agilent BioTek Cytation 5 – manufactured by Agilent, Santa Clara, CA, USA). Phase-contrast images of the platelets were captured using the imaging module of the Cytation 5 at 20× magnification at the end of the experiment.

### 2.3. H-CET Cells

Human corneal epithelial cell lines (H-CET) were seeded at a density of 5000 cell/0.1 mL H-CET media in 96-well plates (clear bottom, NUNC). Following 70% confluency, the cells were labeled with a respective fluorescent ROS detecting dye—MitoSOX Red 1 µM, CellROX Red 2.5 µM or DCFDA 1 µM. The cells were incubated with the ROX indicator for 30 min and then treated with normal plasma or SJS plasma. The fluorescence of the ROS indicators was measured using the Cytation 5 fluorescence plate reader after 1 h.

## 3. Results

IL-1β, IL-6, and RIPK3 are well-established biomarkers that play key roles in various inflammatory diseases. To examine their expression in SJS/TEN patients, LP patients, and normal controls, we conducted the experiments described in the previous section. The findings from these experiments are detailed below.

**Figure 2:**
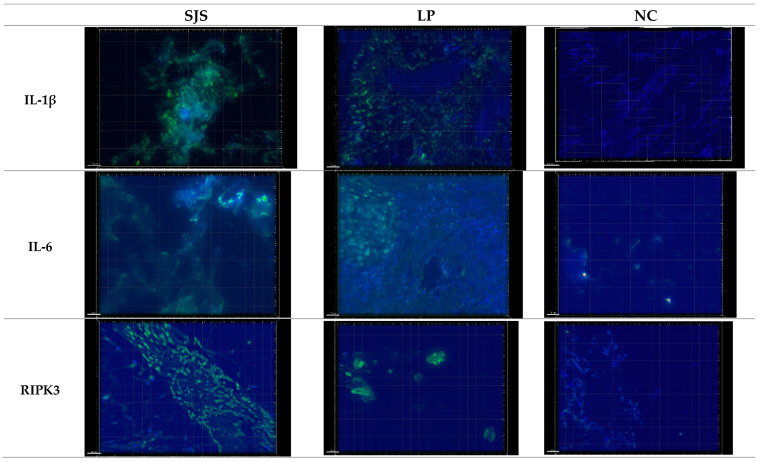
Representative images captured using Deconvoluted Immunofluorescent (DIF) microscopy with a DeltaVision Microscope equipped with a 20× lens and a digital camera. Exposure times were standardized across all samples. Increased expression of IL-1β, IL-6, and RIPK3 was observed in SJS and LP skin biopsy slides compared to NC.

**Figure 3:**
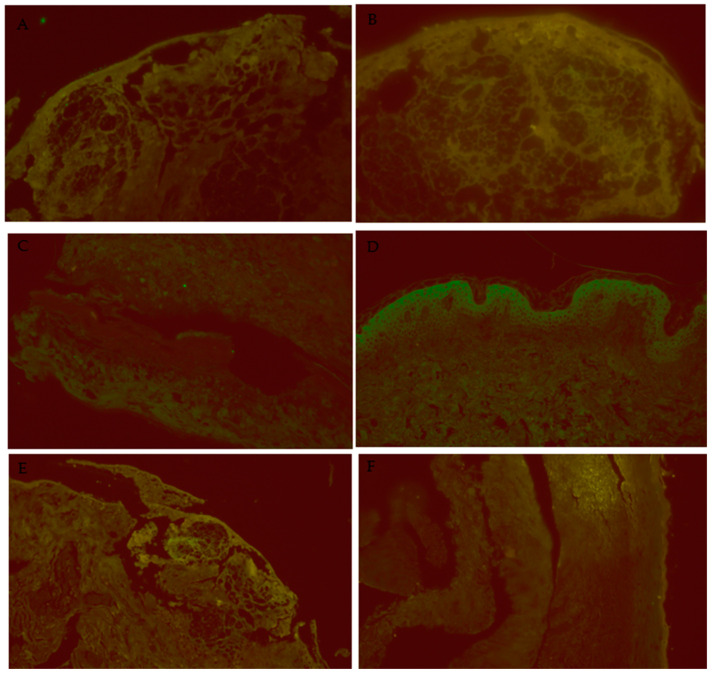
Additional images of DIF miscroscopy. (**A**): SJS/IL-1β/40×, IL-1β is highlighted in the areas of full-thickness epidermal necrosis. (**B**): SJS/IL-6/40×, IL-6 is highlighted in the areas of full-thickness epidermal necrosis. (**C**): SJS/RIPK3/40×, focal staining of RIPK3 is present in the areas of full-thickness epidermal necrosis. (**D**): NC/IL-6/40×, some bright staining is apparent in the upper levels of the epidermis. (**E**): SJS/IL-6/20×, IL-6 is highlighted at the dermal-epidermal junction, where the lichenoid inflammation and interface activity was occurring. (**F**): LP/IL-6/20×, no significant staining is visible in this skin section.

For each of the three experimental conditions, 10 images were captured from skin biopsy slides of SJS/TEN, LP, and normal controls. The average number of puncta per image was analyzed, with all puncta exceeding the background auto-fluorescent intensity when pooled for statistical evaluation. A Kruskal–Wallis test was performed, followed by pairwise comparisons using Dunn’s test with a correction for multiple testing (Figure 2 and Figure 3).

### 3.1. IL-1β Puncta Count and Intensities in SJS/TEN, Lichen Planus, and Normal Control Tissue Samples

A significant difference was observed in the expression of IL-1β within the epithelium across pooled samples from the SJS/TEN patients (average immunofluorescence [IF] intensity: 1348 a.u.), lichen planus (LP) patients (average IF intensity: 1279 a.u.), and normal controls (average IF intensity: 1168 a.u.) (*p* < 0.0001). The SJS/TEN group exhibited a significantly higher IL-1β intensity compared to the LP group. However, no other pairwise comparisons showed significant differences.

**Figure 4:**
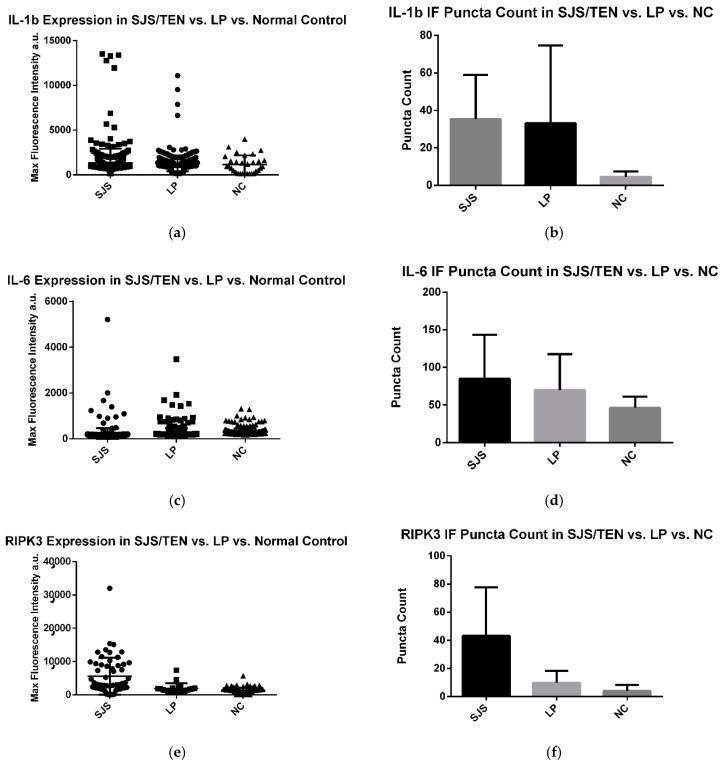
(**a**–**f**) IL-1β, IL-6, and RIPK3 expression in LP, SJS/TEN, and NC skin samples. Immunofluorescent puncta above baseline fluorescence for SJS/TEN patients were counted, pooled, and compared against pooled data from LP and NC. Experimentation revealed a significant difference in the expression of the markers in the skin of SJS/TEN, LP, and NC patients.

Similarly, a significant difference was noted in the average number of IL-1β puncta among the SJS/TEN patients (35 puncta), LP patients (33 puncta), and normal controls (5 puncta) (*p* < 0.0001). The SJS/TEN samples displayed significantly more IL-1β puncta compared to normal controls, but no significant differences were found in other pairwise comparisons (Figure 4).

### 3.2. IL-6 Puncta Count and Intensities in SJS/TEN, Lichen Planus, and Normal Control Tissue Samples

A significant difference in IL-6 expression was observed in the epithelium of pooled samples from SJS/TEN patients (average immunofluorescence [IF] intensity: 299 a.u.), lichen planus (LP) patients (average IF intensity: 241 a.u.), and normal controls (average IF intensity: 204 a.u.) (*p* < 0.0001). The SJS/TEN group exhibited a significantly higher IL-6 intensity compared to both the LP group and normal controls.

In contrast, there was no significant difference in the average number of IL-6 puncta between SJS/TEN patients (85 puncta), LP patients (75 puncta), and normal controls (46 puncta) (*p* = 0.3575).

### 3.3. RIPK3 Puncta Count and Intensities in SJS/TEN, Lichen Planus, and Normal Control Tissue Samples

A significant difference in RIPK3 expression was observed in the epithelium among the pooled samples from the SJS/TEN patients (average immunofluorescence [IF] intensity: 5590 a.u.), lichen planus (LP) patients (average IF intensity: 1979 a.u.), and normal controls (average IF intensity: 1345 a.u.) (*p* < 0.0001). The SJS/TEN group exhibited a significantly higher RIPK3 intensity compared to both the LP group and normal controls.

There was also a significant difference in the average number of RIPK3 puncta between the groups: SJS/TEN patients had 43 puncta, LP patients had 10 puncta, and the normal controls had 4 puncta (*p* = 0.0204). The SJS/TEN group showed significantly more RIPK3 puncta compared to both the LP group and normal controls.

### 3.4. Mitochondrial Functioning Assays

**Figure 5:**
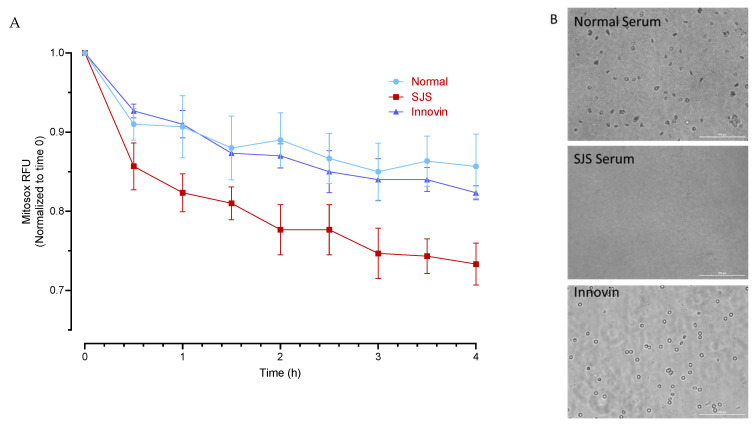
Cytotoxic effect of SJS Plasma on Human platelets. (**A**) Mitosox fluorescence was measured in MitoSOX labeled platelets following treatment with normal plasma, SJS plasma, and Innovin. (**B**) Representative phase contrast images of platelets (20×; Scale bar 100 µm) following treatment with normal and SJS plasma and Innovin.

Platelets:

As observed in the phase-contrast images, SJS plasma significantly reduced the viability of platelets. There was also a significant decrease in MitoSOX fluorescence in platelets following treatment with SJS plasma (Figure 5).

## 4. H-CET

**Figure 6:**
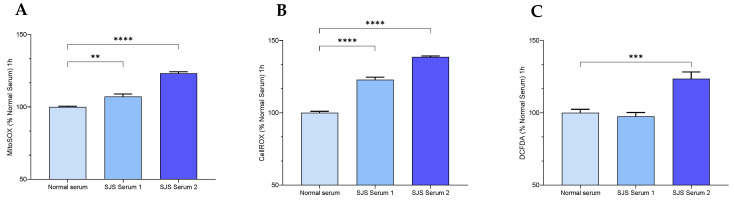
SJS Plasma induces reactive oxygen species in H-CET cells. Cell ROS and Mitochondrial ROS were measured in H-CET cells following treatment with Normal and SJS Plasma. (**A**) Mitochondrial superoxide as measured by MitoSOX Red fluorescence. (**B**) Total cellular superoxide as measured by CellROX Red. (**C**) Total cellular ROS as measured by DCFDA. If a *p*-value is less than 0.01, it is flagged with 2 stars (**). If a *p*-value is less than 0.001, it is flagged with three stars (***). If a *p*-value is less than 0.0001, it is flagged with four stars (****).

**Figure 7:**
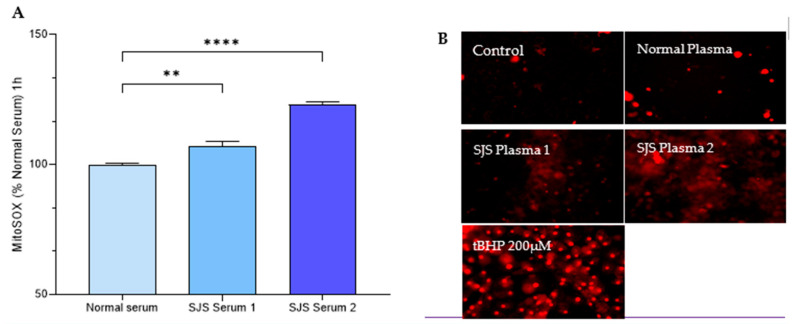
SJS Plasma Induces Mitochondrial ROS in H-CET cells. (**A**) Mitosox Fluorescence was measured in MitoSOX labeled HCET cells following treatment with normal plasma and SJS plasma. (**B**) Representative fluorescence images of HCET cells (20×; Scale bar 100 µm) following treatment with normal plasma, SJS plasma, media and tBHP (positive control). Note: The fluorescent images were captured after 24 h so the data are not that reliable. The images had variable backgrounds, so they are not all accounted for with the same intensity. Images at earlier hours need to be taken for quantitative analysis. If a *p*-value is less than 0.01, it is flagged with 2 stars (**). If a *p*-value is less than 0.0001, it is flagged with four stars (****).

SJS plasma consistently and significantly increased the mitochondrial and total cellular superoxide ions as indicated by a significant increase in MitoSOX Red and CellROX red. However, the effect on total cellular ROS as measured by DCFDA was consistent with normal human plasma from one of the donors (Figure 6 and Figure 7).

## 5. Discussion

Stevens–Johnson Syndrome/Toxic Epidermal Necrolysis (SJS/TEN), collectively referred to as epidermal necrolysis, are rare but highly fatal autoimmune disorders. They cause systemic complications, including skin necrosis and sloughing, rashes, mucous membrane damage, and hematological, ophthalmological, and genitourinary issues. Research into these diseases’ pathogenesis has emphasized the importance of cytokines in their development. Our findings highlight that IL-1β, IL-6, and RIPK3 play pivotal roles in these disease processes, with significantly elevated expression levels observed in the skin of SJS/TEN patients compared to those with lichen planus (LP) or normal controls. Additionally, mitochondrial stress tests showed a marked reduction in the oxygen consumption rate (OCR) of platelets and human corneal epithelial cells (H-CET) exposed to SJS/TEN plasma, indicating significant mitochondrial dysfunction.

Our findings indicate that IL-1β, IL-6, and RIPK3 may play potential roles in the disease process, with significantly elevated expression levels in the skin of SJS/TEN patients compared to those with lichen planus (LP) or normal controls. Mitochondrial stress tests further revealed a marked reduction in the oxygen consumption rate (OCR) of platelets and human corneal epithelial cells (H-CET) exposed to SJS/TEN plasma, signifying substantial mitochondrial dysfunction.

IL-1β, IL-6, and RIPK3 were expressed in all three groups—SJS/TEN, LP, and normal controls—with significantly higher levels observed in the SJS/TEN group. This suggests these cytokines and inflammasomes, which are typically involved in cell death regulation (e.g., pyroptosis) and tissue repair, could play a critical role in epidermal necrolysis [30]. Elevated IL-6 levels in LP patients, consistent with previous studies [31], served as a positive control for the experiments. Although cytokines were elevated in normal controls and LP patients, their expression was markedly higher in SJS/TEN patients, underscoring their relevance to disease pathogenesis.

Despite these findings, the role of IL-1β remains unclear when considered alongside results from Huyen et al. [32], which showed significantly higher IL-1β concentrations in normal control plasma than in SJS/TEN plasma. This discrepancy may arise from IL-1β being released by necrotic keratinocytes during disease progression, with levels diminishing as re-epithelialization occurs. Healthy keratinocytes may also produce and store more IL-1β than necrotic keratinocytes. Further research is needed to determine the timing of IL-1β upregulation.

The upregulation of IL-6 in SJS/TEN patients has been corroborated by multiple studies. Stern et al. observed that elevated IL-6 levels correlate with the extent of cutaneous involvement and clinical course severity [19]. IL-6 has also been implicated in the inflammatory pathogenesis of SJS, which tends to be more severe than TEN. Tear cytokine assessments in chronic SJS patients revealed a significant upregulation of IL-6 compared to controls [33], and its chronic elevation has been associated with corneal and conjunctival epithelial damage [34].

Recent work by Kim et al. [35] supports a key role for RIPK3 in SJS/TEN pathogenesis. The study showed that RIPK3 is significantly upregulated in TEN skin lesions, leading to reactive oxygen species (ROS) generation, mixed lineage kinase-like protein activation, and necroptotic cell death of keratinocytes. These findings suggest that necroptosis effectors, alongside apoptotic pathways, contribute to epidermal cell death in TEN. RIPK3 inhibitors, which are currently being developed in preclinical testing, may offer therapeutic potential for SJS/TEN patients.

Mitochondrial ROS (mtROS) is increasingly recognized as a critical signal for NLRP3 inflammasome activation, which precedes IL-1β production. While ROS from NADPH oxidase has been implicated in inflammasome activation [35], other studies have shown that inhibiting NADPH oxidase does not always affect inflammasome activity [36]. Evidence suggests that mtROS generated by mitochondrial respiratory chain inhibition plays a central role in activating the inflammasome [32,34]. Nakahira et al. demonstrated that dysfunctional mitochondria generate mtROS, which is essential for NLRP3 inflammasome activation [36].

Our results revealed significantly reduced OCR in platelets and cells from SJS/TEN patients compared to normal controls, indicative of mitochondrial damage. This damage likely results in increased mtROS, heightened inflammasome activation, and reduced metabolic function. Given SJS/TEN’s systemic inflammatory nature, these findings highlight mitochondrial dysfunction as a central mechanism in its pathogenesis.

One limitation of our study is the small sample size, reflecting the rarity of SJS/TEN and limited availability of biopsy samples. Larger sample sizes would enhance the validity of these findings. We also recognize the importance of including a primary negative control, where the primary antibodies are omitted while all other conditions, including the addition of secondary antibodies, remain unchanged. This would help confirm the authenticity of the observed staining pattern. Furthermore, correlating cytokine expression with clinical presentation proved challenging due to inconsistencies in documentation across patient records. Standardized measures, such as the Braden scale, total body surface area affected, and detailed visual assessments, could improve future analyses and facilitate exploration of the relationship between cytokine expression and disease severity.

## 6. Conclusions

Our findings demonstrate a statistically significant increase in the expression of IL-1β, IL-6, and RIPK3 in the skin biopsy samples of SJS/TEN patients compared to those of lichen planus (LP) patients and normal controls. These results suggest that these biomarkers may be critical regulators of the pathogenesis of SJS/TEN in the skin.

Additionally, our results indicate that plasma from SJS patients exerts a cytotoxic effect on platelets and induces reactive oxygen species (ROS) production in H-CET cells. Furthermore, we observed that SJS plasma significantly impairs the metabolic function of cells.

## Data Availability

The raw data supporting the conclusions of this article will be made available by the authors on request.
